# **Fibrous and muscular components of the tarsal tunnel roof; a quantitative 3D anatomical study using dissection and a digital microscribe**

**DOI:** 10.1007/s00276-026-03955-2

**Published:** 2026-08-11

**Authors:** Georga K. Bruechert, Casper G. Thorpe Lowis, William H. B. Edwards, Quentin A. Fogg

**Affiliations:** 1 The University of Melbourne, Melbourne, VIC Australia; 2 The Epworth Hospital, Richmond, Melbourne, VIC Australia

**Keywords:** Tarsal tunnel, Tarsal tunnel roof, Flexor retinaculum, Abductor hallucis muscle, Abductor hallucis lateral fascia, Tibial nerve

## Abstract

**Purpose:**

There is no gold standard technique to diagnose and treat tarsal tunnel syndromes. This is due to the lack of consensus on the structures that form the roof of the tarsal tunnel. The flexor retinaculum is consistently stated to form the roof, but there is no clear understanding of the anatomy (borders, spatial relations) of this fibrous tissue. This makes the surgical release inconsistent, and potentially incomplete, when treating tarsal tunnel syndrome.

**Methods:**

The aim of this study was to accurately describe the roof of the tarsal tunnel. Feet of embalmed Body Donors (*n* = 15; mean age = 84.64 ± 11.21 years; F = 7, M = 7; L = 10, *R* = 5) were analysed. They were dissected and modelled in virtual three-dimensional space. The relations and dimensions of all tissues were measured.

**Results:**

The roof was formed by both the flexor retinaculum and the abductor hallucis (AbH) muscle (*n* = 15), and a lateral fascia of the AbH muscle (*n* = 11). The tarsal tunnel roof was consistently formed by both fibrous and muscular tissues.

**Conclusion:**

These data strongly suggest that this unique, multi-tissue organisation of the roof, needs to be further assessed when surgically decompressing the contents of the tarsal tunnel. This may inform more accurate techniques for tarsal tunnel release.

## Introduction

Tarsal tunnel syndrome contributes to pain, decreased function and decreased quality of life, which is experienced by many worldwide [1, 3, 11]. It may evolve due to the compression of the tibial nerve from various causes (e.g., fractures, rheumatoid arthritis, neuroma, a tight flexor retinaculum), with the development of diabetes or, in a majority of cases (12–69%), be caused by unknown aetiologies [1, 3, 8, 10, 11, 17, 23].

Treatment of tarsal tunnel syndrome is most commonly conservative, followed by surgical release [8, 10, 14]. When surgical release is indicated, various approaches are used. These include: blunt dissection between the tibial nerve and the flexor retinaculum; inserting an obturator and cannulated sleeve into the tarsal tunnel; curvilinear incision of the flexor retinaculum; ultrasound-guided or endoscopic-guided incision of the flexor retinaculum and abductor hallucis (AbH) muscle; or full exposure of the tibial nerve via incision of the flexor retinaculum and overlying vessels [3, 5, 8, 14, 17, 23, 27, 29]. With these various approaches and causes of tarsal tunnel syndrome, there are a wide variety of outcomes.

The general form and contents of the tarsal tunnel are accepted, with its shape bounded by the medial malleolus dorsally, the calcaneus plantar-laterally, and the talus laterally [6, 9, 11, 28]. There is no census however on key features, such as all tissues that contribute to the “roof”, the proximal and distal margins of the tunnel, and the attachments of these tissues. The fibrous “roof” of the tunnel, or the medial “wall”, is consistently reported to be defined by the flexor retinaculum, a thick fascia that prevents ‘bowstringing’ of the tarsal tunnel tendons as they cross the talocrural joint into the foot [4, 7–9, 12, 28]. The flexor retinaculum, however, is described in vastly different ways throughout the reviewed literature and there has been no clear description of how it was differentiated from adjacent surrounding soft tissue structures, such as the crural fascia of the leg [7–9, 12, 28, 29]. For example, it has been described to be attaching between the medial malleolus of the tibia and the calcaneus, with either undefined proximal and distal boundaries [3, 23, 28], extending 20 mm either side of malleolar-calcaneal line [7], extending distally to the distal border of a 10 mm wide malleolar-calcaneal line [28], or overlapping with the crural fascia [12]. Methodological differences between studies may have contributed to the lack of consensus, with dissection, imaging and other techniques (macro-sections, plastination, etc.) used variably [7, 9, 12, 14, 18, 20, 26]. With a lack of clarity and uniformity in the protocols used, reproducible results are not able to be obtained.

The AbH muscle and/or its lateral fascia have also been suggested in few studies to contribute to the tarsal tunnel “roof” [3, 11, 20, 29] or at least compress the tibial nerve and/or its branches [3, 8, 11, 25]. It is not clear whether the AbH consistently contributes to the “roof” and, therefore, whether it should always be considered when treating tarsal tunnel syndrome, and if so, how it can be without interrupting its own structural integrity. This may depend on where within the tarsal tunnel, compression is occurring. For example, whether inconsistent points of reference and terminology may add to the lack of clarity. Terms that clearly describe what the structure/s are doing should be used. In dissection studies, vague definition of specific tissues was another potential cause of the discrepancy between results [7, 12]. An accurate and reproducible investigative approach to the tarsal tunnel is therefore needed.

A clear understanding of the structure and arrangement of the tarsal tunnel is essential for adequate surgical release. The high incidence of poor clinical outcomes and revision may be attributable to incomplete sectioning of surrounding soft tissues that may be compressing the vessels and/or nerves, or sectioning of the wrong structures [1, 3–5, 14, 15, 17, 23]. Clear and reproducible anatomical data may inform the localisation and extent of sectioning required to provide adequate release of tarsal tunnel compression. This study therefore aims to describe the “roof” of the tarsal tunnel using a combination of dissection and three-dimensional modelling.

## Materials and methods

Feet from embalmed (Genelyn Anatomical Series, Australia) Body Donors (*n* = 15; mean age = 84.64 ± 11.21 years; female = 7, male = 7; L = 10, *R* = 5) were examined. All Body Donors were obtained from the University of Melbourne Body Donor Program. This study was carried out in accordance with *Part IV of the Human Tissue Act (1982)* and with local ethics approval (project ID: 12292).

Each specimen was pre-screened by computed tomography (CT) imaging (Siemens Biograph128 mCT, Siemens Healthcare Pty Ltd). Those with abnormalities that would influence this project (previous surgery, tarsal tunnel or plantar foot deformation) were excluded (*n* = 0). The DICOM data were segmented to allow the production of 3D skeletal models, used as a base for visualisation of soft tissue models.

Using standard surgical instruments, the skin was removed over the medial aspect of the foot, revealing the superficial tissues 100 mm proximal and distal to the medial malleolus. A fascicular dissection approach was applied throughout: following the individual structural elements of a tissue (e.g., fascicles for ligaments, tendons, etc.), rather than assumed structural boundaries (e.g., the borders of a ligament). A surgical microscope (Wild Heerbrugg M651, Switzerland) was used to observe these tissues under 7-36x magnification, as required. This approach minimised displacement of tissues by not releasing connective tissues at the margins of individual structures until each layer was photographed and modelled. Fascicular tissues were followed until they either terminated in a clear attachment, or until they were not discernible from adjacent tissues.

Non-fascicular tissues (e.g., fine fascia composed of irregular connective tissue) were retained until any neighbouring tissues in the same plane had been identified and recorded. Each stage of dissection was photographed using a digital camera (EOS 5D Mark III, Canon, Japan), and two-dimensional (2D) reconstructions (non-volumetric) were created for each structure of interest prior to further dissection.

For each layer of dissection, a 2D planar model of the medial surface of each of the structures of interest was reconstructed in a virtual 3D environment using a digital microscribe (MicroScribe G2X, Immersion Corp., USA) and Rhinoceros 4.0 Software (Robert McNeel and Associates, USA). The microscribe registered a single point in three-dimensional space, as determined at the tip of its stylus and at the direction of the user, through triggering the device (keyboard or foot pedal). Registering a series of points along the surface of a structure created a line in virtual space.

Fascicular tissue is especially suited to such modelling as points can be registered along each fascicle, or a representative number of fascicles. With sufficient lines created across each surface of interest, a virtual surface was “lofted” over the lines to create the virtual planar 2D surface model of each structure. Every structure was modelled in triplicate, with each modelling sequence done at different times, allowing accuracy and reproducibility of the modelling to be assessed.

The complete model of dissected layers facilitated unique visualisation of their relations. This allowed clear representation of the tissues, irrespective of layer, that contributed to the roof, and which structures contributed to the definition of the proximal and distal margins of the tunnel. These can be easily visualised after dissection, as each tissue layer can be turned on/off in the software.

To validate the accuracy of the techniques used, comparisons were made between the rounds of modelling for each structure. A one-way ANOVA was carried out to compare each round, with a significance of 0.05 used throughout all statistical analysis. Where no significant difference was found between rounds of modelling, the tested model was deemed suitably accurate and representative of the real specimen.

## Results

**Fig. 1:**
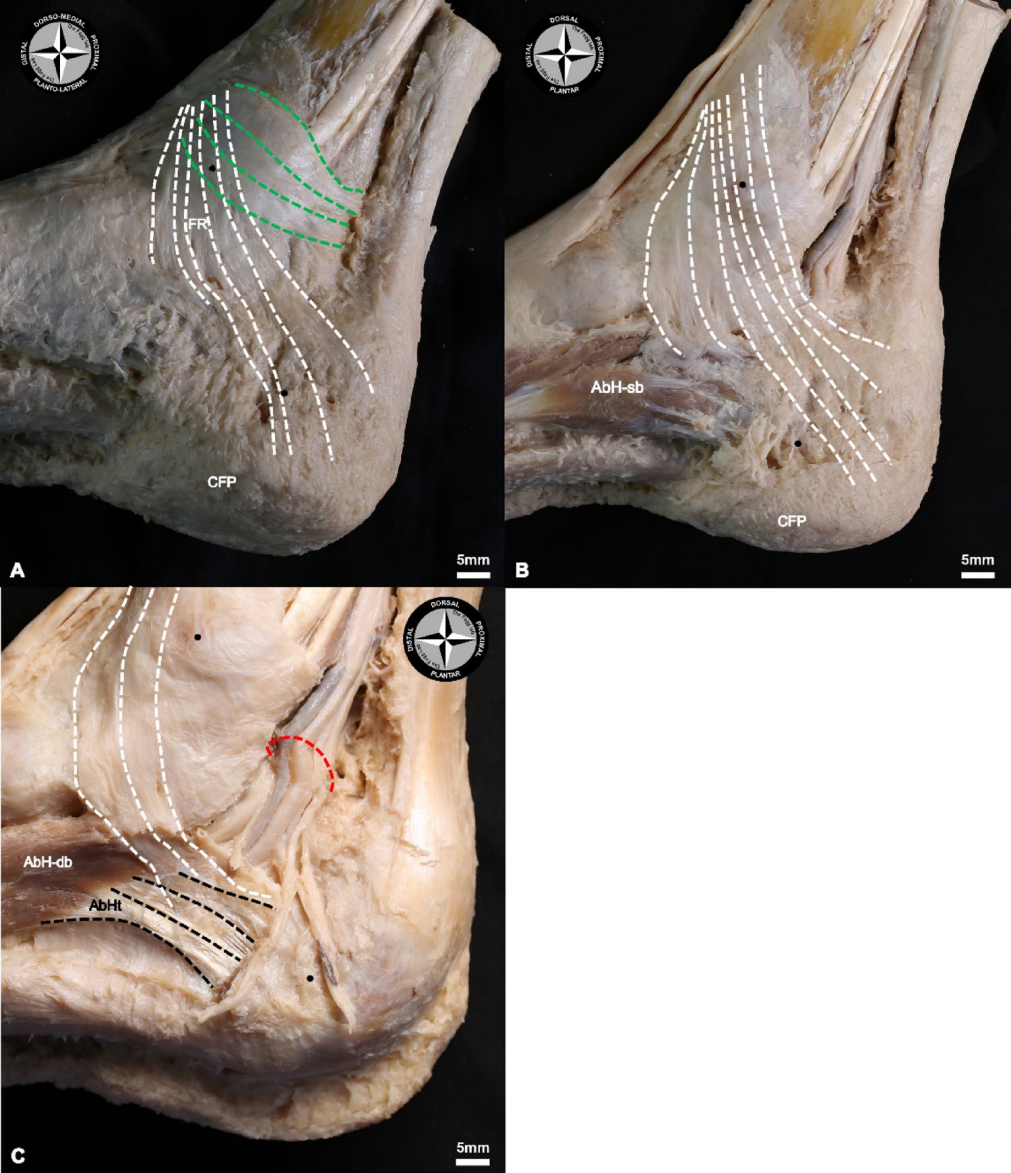
An angled medial view of the roof of the tarsal tunnel. **A** The borders of the flexor retinaculum (FR) were determined according to the orientation of its fascicles (dashed white lines) continuing from the medial malleolus and plantarly towards the calcaneus, blending with the calcaneal fat pad (CFP). There were fascicles of the crural fascia (partially removed) that overlapped with the flexor retinaculum to course from the medial malleolus to the calcaneal tendon (dashed green lines), which were not considered as part of the flexor retinaculum. Black dots: Pins A and B. **B** The fascicles of the flexor retinaculum (dashed white lines) were also seen to slightly overlie the abductor hallucis muscle (AbH) superficial belly (AbH-sb); the more distal fascicles terminated along the dorsal border of the muscle; the more proximal fascicles continued to the calcaneus and CFP. **C** Lateral/deep to the AbH-sb, a thick tendon of the AbH (AbHt: dashed black lines) was present, attaching the proximal end of the deep belly of the AbH muscle (AbH-db) to the calcaneus. A few of the more distal fascicles of the flexor retinaculum (dashed white lines) continued to attach and blend into the AbHt. This AbHt was evidently overlying the posterior tibial artery and its venae comitantes (dashed red semi-circle)

The medial roof of the tarsal tunnel was formed by both the flexor retinaculum and the abductor hallucis (AbH) muscle in all cases (*n* = 15; 100%). Fascicles of the flexor retinaculum were first identified slightly distal and dorsal to the medial malleolus (Fig. 1A). These fascicles consistently continued from this region, superficial to the medial malleolus, before projecting off of the plantar-proximal margin of the malleolus to form the true retinaculum. The most dorsal of these fascicles were interdigitated with crural fascia fascicles (Fig. 1A). The fascicles of the crural fascia diverged from the retinacular fascicles and coursed posteriorly rather than plantarly, and did not attach between the medial malleolus and the calcaneus. The crural fascicles were therefore not considered part of the retinaculum, giving the retinaculum a distinct proximal, fascicular margin. The longest fascicles of the retinaculum were traced in a plantar and posterior direction to where they consistently terminated in the fibrous tissues of the calcaneal fat pad (Fig. 1). The most distal retinacular fascicles superficially blended into the medial fascia of the foot (*n* = 14; 93%). In some cases (*n* = 8; 53%), fascicles of the flexor retinaculum were also attached to the dorsal margin of the AbH muscle (Fig. 1B). The consistent association with the AbH muscle made the determination of the distal margin of the tunnel based on retinacular tissue alone unlikely.

Upon removal of the fascia overlying the medial/superficial surface of the AbH muscle, the fascia continued plantarly. At the plantar border of the AbH muscle, the fascia met with the medial border of the thick plantar fascia, the plantar aponeurosis, partially encasing the AbH muscle.

After the reflection of its medial fascia, two proximal continuations of the AbH muscle were identified and distinguishable from one another (*n* = 12; 80%). In the remaining specimens (*n* = 3; 20%) the number of AbH muscle bellies and their attachments were not clear. In the former instance, the medial/superficial muscle fascicles interdigitated with retinacular fascicles and either attached into the calcaneus directly as muscular fascicles (*n* = 4; 27%), or with the addition of thin tendonous fascicles (*n* = 8; 53%). Careful removal of these fascicles revealed a deeper muscle belly in one case (7%), whilst in a majority of cases (*n* = 11; 73%), a deeper, tendinous continuation of the muscle was present. This tendon was sometimes present on the deep surface of the whole AbH muscle (*n* = 3; 20%), which could not be separated. When present, this proximal AbH tendon attached further proximally/posteriorly to the calcaneus (Fig. 1C). This tendon sometimes received fascicles from the retinaculum (*n* = 7; 47%; Fig. 1B, C).

With the removal of the AbH muscle, the plantar attachment of the medial AbH muscle fascia was explored (*n* = 14; 93%). Plantarly, where the medial AbH fascia met the medial border of the plantar aponeurosis, the former was identified to attach into the plantar border of the deep AbH tendon (*n* = 7; 47%), blend in with a thicker fascia deeper/lateral to the AbH muscle (*n* = 2; 13%), or to have no clear attachments (*n* = 5; 33%). Proximally, fascicles of the medial AbH fascia continued with the calcaneal fat pad or, those directly along the surface of the AbH muscle, attached to the calcaneal bone.

The AbH muscle, and particularly the proximal tendon, consistently and considerably contributed to the “roof” over the tarsal tunnel. The most plantar margin of the proximal AbH tendon was therefore considered the distal margin of the tunnel.

**Fig. 2:**
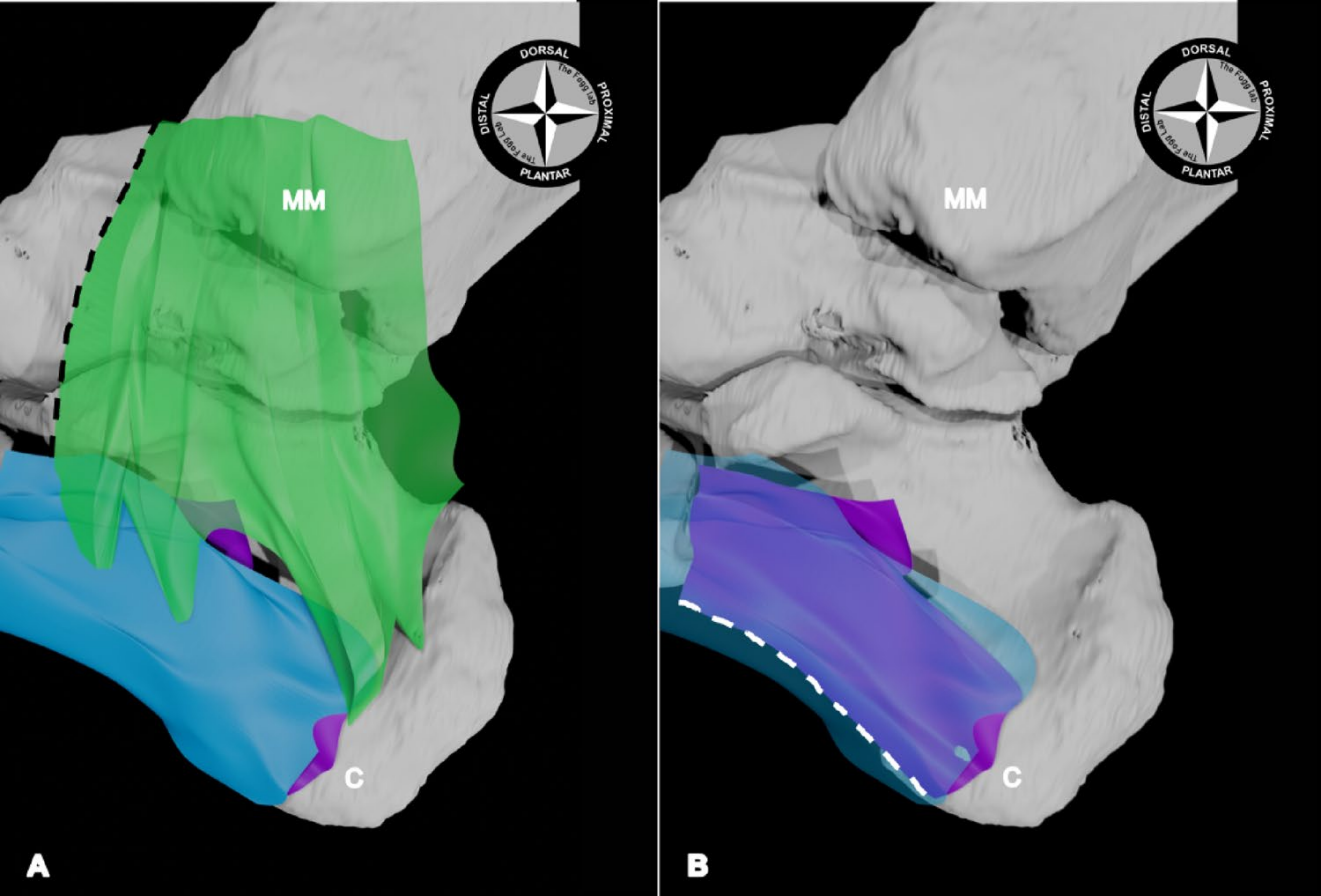
A 3D model of a single specimen, representing the structures that may contribute to the roof of the tarsal tunnel across all specimens. **A** The flexor retinaculum (green) always formed the most proximal medial roof of the tarsal tunnel (black dashed line: distal border of flexor retinaculum). It frequently intersected or attached into the abductor hallucis (AbH) muscle (blue), with the AbH tendon (purple) closely associated. **B** The AbH muscle (light blue) and tendon thereby formed the distal medial roof of the tarsal tunnel (white dashed line). MM: medial malleolus; C: calcaneus

Digital modelling of each layer of dissection allowed for stacked visualisation of all layers. The combined view (Fig. 2) of the superficial and deep layers of the roof, each with proximal and distal components, allowed for clear determination of the entrance and exit to the tarsal tunnel. The modelling demonstrated that the proximal margin was determined by the distinctly fascicular flexor retinaculum (*n* = 15; 100%) as it coursed from the tissues superficial to the medial malleolus to the calcaneal fat pad (Fig. 2A). The distal margin was determined by the deeper layer of the roof, by the plantar margin of either the AbH muscle (*n* = 1; 7%) or the proximal AbH tendon (*n* = 14; 93%; Fig. 2B).

The modelling of structures was assessed for accuracy and reproducibility. The areas/volumes between triplicated rounds of all modelled structures showed no significant difference (*p* > 0.05), suggesting that all of the data presented were accurate and reproducible.

## Discussion

A clear understanding of the boundaries and constituent tissues of the tarsal tunnel roof is essential for accurate surgical release of compressed tunnel contents [12, 14, 21, 23]. Previous studies typically only considered the flexor retinaculum to be forming the roof, and did not clearly define the borders of the flexor retinaculum [7, 11, 25]. The borders of the tarsal tunnel had also been suggested numerically, 20 mm on either side of the medial malleolar-calcaneal line, perhaps in deference to the difficulty in identifying distinct tissue margins [13]. Such difficulty may have been borne by dissection methodologies that lacked criteria for tissue selection.

The current study employed a fascicular dissection strategy that included a clear decision-making process at the tissue level. It is hoped that this approach will provide subsequent studies with a reproducible methodology that will help expand understanding of this area. Using this approach, the current study demonstrated that the medial roof was consistently formed by both the flexor retinaculum and either the AbH muscle or its proximal tendon. A limited number of previous studies support this description, with some limiting the roof to the flexor retinaculum [7, 11, 12, 20, 21, 29]. Others suggest that the AbH muscle may contribute to nerve compression, but do not include the muscle as a part of the roof [3, 8, 15, 25, 30]. A clear point of reference and clear terminology is required to define the boundaries of the tarsal tunnel.

In the current study, the boundaries of the tarsal tunnel were consistently determined by the arrangement of the roof. The proximal border was composed only of flexor retinaculum fascicles, which distinctly diverged from crural fascia fascicles. These have previously been considered indistinguishable from one another, unless the retinaculum demonstrated a denser consistency through palpation [18]. Reports of proximal branching of the tibial nerve therefore should be considered a pre-tunnel bifurcation [2, 7, 13, 18, 28]. The crural fascia may still be thick, potentially contributing to compression, so this tissue should be considered in patients with pre-tunnel branching of their tibial neurovasculature. Further investigation, particularly via ultrasound and/or exoscope, could determine the accuracy of identifying the distinction between the flexor retinaculum and crural fascia in a clinical setting [14, 19, 20].

The distal border of the tunnel was consistently determined by the AbH muscle or its proximal tendon. The proximal part of the AbH muscle was either muscular to its proximal attachment to the calcaneus or was attached to the calcaneus via a prominent tendon. This differed from most previous descriptions, which have not described the attachments of the AbH muscle clearly, nor discussed its contribution to the medial roof of the tarsal tunnel [14, 17, 18]. The current data strongly suggest that the proximal AbH muscle should be considered in the treatment of tarsal tunnel compression. Lack of consideration of this muscle may contribute to the poor post-operative outcomes of tarsal tunnel release [1, 15, 30].

The current data also suggest layering of the tarsal tunnel roof, with the retinaculum more prominent superficially/medially and proximally, and the AbH muscle and tendon more prominent deep/laterally and distally (Fig. 2). This was not represented within the literature. The change in tissue forming the roof, from the passive restraint conferred by the retinaculum, to the dynamic restraint of the muscle, suggests changing biomechanical conditions within the tunnel. Further study is needed to explore the intra-tunnel supports, how they change throughout the length of the tunnel, if they contribute to the organisation of the plantar components of the foot, and ultimately if their surgical ‘release’ to decompress underlying neurovascular structures could disrupt their biomechanical support to the foot.

The fascia around the AbH muscle was consistently connected to the medial border of the plantar aponeurosis. The reviewed literature did not provide a clear description of this relationship, nor was it consistently noted; mechanical changes in the plantar aponeurosis may therefore indirectly contribute to tarsal tunnel compression [4]. These connections also suggest a role of these tissues in the compartmentalisation of the plantar foot, which warrants further study. Such information may provide more insight into an anatomical basis for more accurate tarsal tunnel release and inform how tarsal tunnel conditions may influence plantar foot dysfunction.

There were a number of limitations to the present study. The detailed process of staged dissection, intervened by multiple rounds of digital modelling, provided high-quality, reproducible data, but strongly limited the number of specimens that could be investigated, and the range of modalities used. Future studies, should resources permit, may combine the current dissection/modelling approach with clinical imaging. In particular, combining the current approach with ultrasound imaging will provide data more readily applied in a clinical setting. The current study demonstrated more extensive fascicular connections than previous studies. As previous dissection studies typically lacked a clear indication of the decision-making process involved in tissue retention (what tissues were kept and what tissues were removed), it is recommended that future studies follow a similar method to the current study. This will facilitate comparison between studies to an extent that was not possible with the current literature.

Tarsal tunnel syndromes are common issues. As the major conduit to the plantar aspect of the foot, impaction of the tarsal tunnel may further contribute to plantar compartment syndromes [22]. These syndromes may develop at the level of the tarsal tunnel, suggesting that the extent and construct of the medial roof may, in turn, contribute to their development. In particular, variation in the status of the AbH muscle may contribute to intra-tunnel compression, such as when the muscle is either hypertrophied or injured [8]. Relations of the tarsal tunnel roof to the calcaneus should also be considered; with retinacular and muscular connections to the calcaneal fat pad, and tendinous or muscular attachments to the calcaneus, injuries to the heel may also alter conditions in the tarsal tunnel. These structures should therefore be considered during the repair of calcaneal fractures, as well as during surgical procedures involving calcaneal osteotomies and tendon transfers at the hindfoot [16, 24]. Neurovascular structures coursing to and from the calcaneal region may also be influenced by relations to the multi-tissue tarsal tunnel roof, especially if they traverse the roof to enter/exit the tunnel. Further study is required to determine these relations, which may further inform the management of injuries in this broader area.

## Conclusion

The current study has demonstrated the unique, multi-tissue organisation of the tarsal tunnel roof. Relations to surrounding tissues have been demonstrated, suggesting a broader range of injuries that may contribute to the compression of tarsal tunnel contents. These data will provide a basis for further investigation into a clear anatomical basis for tarsal tunnel release, and how this procedure may be optimised. These data, combined with more detail of the intra-tunnel organisation and relations, may inform advances in tarsal tunnel release techniques, improving post- operative outcomes.

## Data Availability

The data that support the findings of this study are not publicly available due to ethical restrictions. Access may be granted upon reasonable request via the corresponding author, subject to institutional approval and ethical compliance.
